# The Treatment Response and Safety Profile for Mirabegron in Women With Overactive Bladder and Hypertension

**DOI:** 10.7759/cureus.84855

**Published:** 2025-05-26

**Authors:** Jean Ee Neo, Mark K Lui, Yvonne W Wong, Jill C Lee

**Affiliations:** 1 Yong Loo Lin School of Medicine, National University of Singapore, Singapore, SGP; 2 Obstetrics and Gynaecology, KK Women’s and Children’s Hospital, Singapore, SGP; 3 Gynaecology-Oncology, Singapore General Hospital, Singapore, SGP; 4 Urogynaecology, KK Women’s and Children’s Hospital, Singapore, SGP

**Keywords:** beta-3-agonist, bladder training, hypertensive patients, menopause, mirabegron, mixed urinary incontinence, overactive bladder, stress urinary incontinence, urge urinary incontinence, β3-adrenergic receptor agonist

## Abstract

Background and objective

Mirabegron, an established treatment for overactive bladder (OAB), is contraindicated in patients with uncontrolled hypertension. Inevitably, there is concern among clinicians and patients regarding its use in patients with well-controlled hypertension. In this study, we aimed to investigate the efficacy of mirabegron in patients with well-controlled hypertension, as well as its adverse effects.

Methods

This retrospective study involved women with well-controlled hypertension who used mirabegron for the treatment of OAB between August 1, 2017, to July 31, 2020. Medical notes were reviewed from the time of initiation of mirabegron up to 12 months post-treatment. The indicators of response measured were frequency of maturation and nocturia. The sense of urgency and urge incontinence were assessed as well. Safety of the drug was evaluated by documenting the number of hypertensive episodes and recording side effects such as dry mouth, dry eyes, headache, and rashes.

Results

Forty-six patients with OAB and hypertension were included in the study. Of them, 38 patients (73.6%) had improvement in OAB symptoms following mirabegron therapy. Median urinary frequency improved from 1.5 hours to two hours after mirabegron treatment. Twenty-three of the patients stopped experiencing nocturia one month after starting mirabegron. Sixteen patients (76.2%) experienced improvement in urge symptoms while 19 patients (86.4%) experienced improvement in urge incontinence. Of note, 38 patients (78.2%) did not experience any side effects at all, while a small proportion of the patients (n=4, 8.7%) experienced anticholinergic side effects. Three patients (6.5%) experienced an increase in blood pressure after mirabegron use; no additional anti-hypertensive agents were used as mirabegron was stopped once an increase in blood pressure was noted.

Conclusions

Mirabegron is efficacious in improving OAB symptoms. It is well tolerated by hypertensive patients and may be used in the long term for symptom control. Home blood pressure monitoring may aid in the earlier detection of worsening control in the small segment of patients in whom mirabegron is not suitable.

## Introduction

Overactive bladder (OAB) is a highly prevalent condition affecting about 400 million people worldwide [1], resulting in symptoms like urgency, frequent urination, and, in some cases, urgency urinary incontinence. The prevalence of the condition rises with increasing age, affecting 30-40% of individuals aged over 75 years [2]. The initial management involves lifestyle modifications, bladder training, and antimuscarinic medications. However, antimuscarinics can lead to side effects such as dry mouth, constipation, and cognitive decline, especially in older adults, contributing to a high discontinuation rate [2]. Traditional anticholinergic drugs, used for many years to treat OAB, are linked to cognitive impairment, dementia, cardiovascular risks, and increased mortality [3]. The cumulative anticholinergic burden, often worsened by polypharmacy, poses long-term risks.

Mirabegron is the first β3-adrenoceptor agonist approved for the treatment of OAB. It works by stimulating β3 receptors in the bladder, causing detrusor muscle relaxation, which improves bladder storage capacity without impairing voiding contractions [4]. This reduces involuntary bladder contractions that cause OAB symptoms. Mirabegron offers a safer alternative for patients intolerant to antimuscarinics, especially the elderly, thanks to fewer cognitive side effects and minimal impact on other medications [5,6]. Its effectiveness and safety make it an attractive option in the OAB treatment pathway.

A cross-sectional study across three Asian countries reported an OAB prevalence of 22.1% among Asian women, rising from 10.8% in those aged 40-44 to 27.9% in those over 60 years [7]. This increase in prevalence correlates with age, with hypertension being a common comorbidity in these patients [8]. However, mirabegron is contraindicated in patients with uncontrolled hypertension (defined as systolic blood pressure ≥180 mmHg or diastolic blood pressure ≥110 mmHg) due to its potential to increase heart rate and blood pressure [9]. This study aimed to evaluate the efficacy of mirabegron in women diagnosed with OAB and well-controlled hypertension and to assess any potential associated adverse effects in this population.

## Materials and methods

Study design

This single-centre retrospective clinical study was conducted at KK Women’s and Children’s Hospital, Singapore, focusing on patients who began mirabegron therapy for OAB between August 1, 2017, and December 31, 2020. Subjects were identified from the hospital’s pharmacy drug database and approached for consent to participate in the study. We reviewed medical records of study participants from the start of mirabegron treatment up to 12 months post-treatment. We assessed lower urinary tract symptoms -such as frequency, urgency, nocturia, stress urinary incontinence (SUI)

, and urgency urinary incontinence - at follow-up visits scheduled at one month, three to four months, six to seven months, and 9-12 months. Blood pressure measurements were recorded before starting mirabegron and at each follow-up visit. We also reviewed reasons for treatment discontinuation and any adverse effects reported by participants. Exclusion criteria included (i) patients who had received mirabegron before the study period, (ii) patients on dual pharmacotherapy for OAB, (iii) patients under 21 years or over 90 years of age, and (iv) patients who did not have pre-existing hypertension.

Evaluation of blood pressure

Blood pressure was assessed using an automated oscillometric upper arm monitor. During the procedure, the patients were placed in a quiet room at a comfortable temperature; they were advised to avoid talking during the procedure. During the procedure, we ensured that the patients were seated on the chair with their feet flat on the floor.

Statistical analyses

Data, except for age, are presented as medians due to the skewed distribution of variables such as frequency, urgency, nocturia, SUI, and urgency urinary incontinence. Statistical analyses were conducted using Microsoft Excel software (Microsoft Corporation, Redmond, WA).

## Results

Patient characteristics

**Figure 1:**
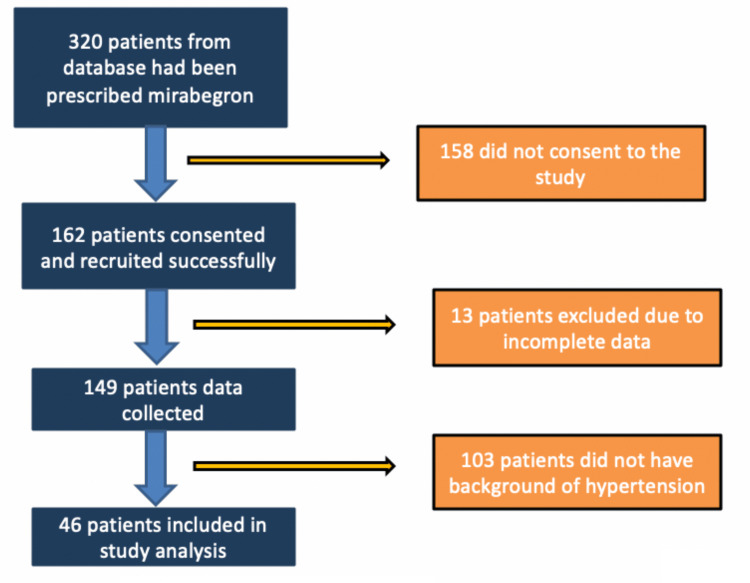
Patient selection process

Of the 320 patients screened from the hospital pharmacy drug database, 158 were excluded since they did not consent to participate in the study. A further 103 were excluded because they did not have pre-existing hypertension. The study finally included 46 patients, all of whom had hypertension and were on antihypertensive medications (Figure 1). Patient characteristics are presented in Table 1.

**Table 1:** Patient characteristics (n=46) ^*^Some patients had more than 1 medical condition or had undergone more than one surgery. ^**^In this study, premenopausal women were not offered topical estrogen PFR: pelvic floor repair; SD: standard deviation; SSF: sacrospinous fixation

Variables	Values
Age, years, median (range)	65.6 (44–93)
Race, n (%)	
Chinese	40 (87.0)
Malay	3 (6.5)
Indian	2 (4.3)
Others: Filipino	1 (2.2)
Parity, n=45, mean ± SD (range)	2.1 ± 1.5 (0–8)
Parity, n=45, n (%)	
<3	32 (71.1)
≥3	13 (28.9)
Nulliparous, n=45, n (%)	7 (15.6)
Menopause, n (%)	43 (93.5)
Medical history^*^, n (%)	
Diabetes mellitus	13 (28.3)
Hypertension	46 (100)
Lipids	26 (56.5)
Cardiac conditions	4 (8.7)
Thyroid	3 (6.5)
Gastritis	1 (2.2)
Musculoskeletal disease	5 (10.9)
Cancer	1 (2.2)
Glaucoma	1 (2.2)
Autoimmune condition	2 (4.3)
Myasthenia gravis	1 (2.2)
Hepatitis B	1 (2.2)
Dementia	1 (2.2)
Fibroid	1 (2.2)
Gout	1 (2.2)
Stroke	2 (4.3)
Neurofibromatosis	1 (2.2)
Cyst/ovarian cyst	2 (4.3)
Renal calculi	1 (2.2)
Recurrent urinary tract infection	1 (2.2)
Surgical History^*^, n (%)	
Hysterectomy	6 (13)
PFR/Restorelle mesh/SSF/cystoscopy	1 (2.2)
Surgery for colon cancer	1 (2.2)
Spine surgery	1 (2.2)
Orthopaedic procedure	1 (2.2)
Use of topical estrogens, n=43^**^, n (%)	
Vagifem	8 (18.6)
Premarin	22 (51.2)
Nil	14 (32.6)

As detailed in Table 2, the mean age of participants was 65.6 ± 11.0 years, with the majority being of Chinese race (n=40, 87%). At the time of this study, 93.5% of patients were menopausal. In addition to OAB and hypertension, patients had other cardiovascular risk factors, including diabetes (n=13, 28.3%), hyperlipidemia (n=26, 56.5%), and cardiac conditions (n=4, 8.7%). Some patients had medical conditions which relatively contraindicated for anticholinergic use, such as glaucoma (n=1, 2.2%), myasthenia gravis (n=1, 2.2%), and dementia (n=1, 2.2%). Of the menopausal women, 30 (67.4%) patients were prescribed topical vaginal estrogen.

**Table 2:** Treatment outcomes (n=46) SD: standard deviation

Variable	Before treatment	After treatment
Duration of treatment, months, mean ± SD (range)	5.7 ± 6.2 (0.2–24)	5.7 ± 6.2 (0.2–24)
Frequency, hours, mean ± SD (range)	N=43 [1.7 ± 0.9 (0/5-3.5)]	N=22 [2.1 ± 0.9 (1-3.5)]
<1	0.5 ± 0	0
1–<2	1.2 ± 0.2	1.1 ± 2.2
2–<3	2 ± 0.2	2.2 ± 0.3
3	3 ± 0	3 ± 0.2
>3	3.5 ± 0	3.5 ± 0
Frequency, hours, median, (range)	1.5 (1–2)	2 (1–2)
Nocturia, times	N=41	N=23
Mean ± SD (range)	2.2 ± 1.2 (0.5–5.5)	2.0 ± 0.9 (0.5–3.5)
Nocturia, times, n (%)	N=41	N=23
<1	6 (14.6)	2 (8.7)
1–<2	10 (24.4)	8 (34.8)
2–<3	9 (22.0)	6 (26.1)
3	14 (34.1)	5 (21.7)
>3	1 (2.4)	2 (8.7)
Nocturia, times, median (range)	2 (1–3)	2 (1–2.5)
Urgency, n (%)		N=21
Improved		16 (76.2)
Worsened		0
No change		5 (23.8)
Urge incontinence, n (%)		N=22
Improved		19 (86.4)
Worsened		0
No change		3 (13.6)

Efficacy of mirabegron for OAB symptoms

**Figure 2:**
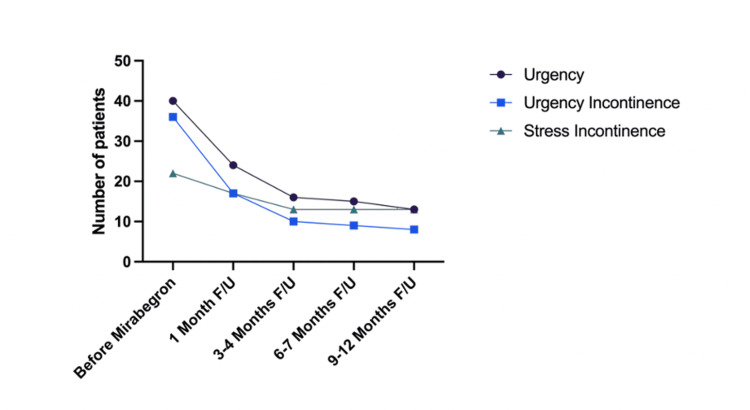
Patients who experienced symptoms of urgency, urgency incontinence, and stress incontinence

Table 3 presents the data on the efficacy of mirabegron for OAB symptoms. The average duration of treatment was 5.7 ± 6.2 months. After treatment, mirabegron was shown to be curative in 29.4% of cases. The drug showed considerable potential by significantly improving symptoms, as 73.6% of patients (n=38) reported an improvement in OAB symptoms. Median urinary frequency improved from 1.5 hours to two hours. Half of the patients no longer experienced nocturia one month after starting mirabegron, with the ideal therapeutic effect achieved by three to four months (Figure 2). Of note, 29.4% of these patients (n=10) stopped mirabegron due to limited effectiveness, with a median time to stop at the 1.6-month mark. This is before our observed ideal duration for optimal therapeutic effect of three to four months. In the whole cohort, 21 patients experienced symptoms of urgency and 22 had symptoms of urge incontinence. After the initiation of mirabegron, patients presenting with urinary urgency (n=16, 76.2%) reported symptomatic improvement. Similarly, in the cohort experiencing urge incontinence, 19 (86.4%) demonstrated clinical resolution. Additionally, of the nine patients who initially experienced stress incontinence, five (55.6%) reported improvement in stress incontinence. Despite these improvements, only 10 patients (21.7%) experienced complete resolution of OAB symptoms.

**Table 3:** Adverse effects from treatment (n=46) ^*^Some patients may have more than one reason for discontinuation of mirabegron OAB: overactive bladder

Parameters	N (%)
Side effects experienced	
Dry mouth/dry eye/constipation/giddiness/blurred vision/palpitation	4 (8.7)
Rashes	1 (2.2)
Headache	2 (4.3)
Increase in blood pressure	3 (6.5)
None	36 (78.2)
Reasons for discontinuation^*^	
Side effects	7 (15.2)
Cost	5 (10.9)
Ineffective	9 (19.6)
Cure	10 (21.7)
Switch to other OAB drugs (e.g., Detrusitol, Vesicare, desmopressin)	1 (2.2)
Not keen to continue	1 (2.2)
Operation	1 (2.2)
Pregnancy	1 (2.2)

Side effects and follow-up

Three patients (6.5%) reported an increase in blood pressure after starting mirabegron. The drug was otherwise well tolerated by the majority of patients (n=36, 78.2%), who experienced no side effects. However, a small percentage reported adverse effects, including headaches (n=2, 4.3%) and rashes (n=1, 2.2%). Of note, 8.7 % experienced anticholinergic side effects (n=4, 8.7%).

A total of 36 patients had discontinued the drug, with nine patients discontinuing as they reported no effect on their symptoms (n=9, 26.5%), with a median time of 1.3 months. An equal number reported resolution of symptoms as their reason for discontinuing (n=10, 29.4%), with a median time to discontinuation of 5.9 months.

## Discussion

The findings of this retrospective study demonstrated that mirabegron is effective in managing OAB symptoms, aligning with findings from larger clinical trials that confirm its safety and efficacy [10,11]. Our study showed that although the majority of patients did not experience significant changes in blood pressure, a minority exhibited elevated blood pressure readings (>140/90 mmHg) following treatment. This underscores the need for cautious monitoring of blood pressure even in patients with well-controlled hypertension who are prescribed mirabegron. While mirabegron remains relatively safe for patients with known hypertension, regular blood pressure assessments are advisable to detect and manage potential increases. A home-based blood pressure monitoring should be recommended in this case, at least once per week, in hypertensive patients, according to local guidance issued by the Singapore Heart Foundation.

A meta-analysis of 52 trials that randomly allocated patients to home blood pressure monitoring (HBPM) or standard clinic-based monitoring found significant benefits associated with home-based monitoring. There was a greater decrease in blood pressure by 3.9/2.4 mmHg at six months in the HBPM arm [12]. This would also mitigate white coat hypertension, aid in the assessment and identification of true hypertensive adverse effects of mirabegron, and contribute to patient medication compliance. This recommendation is consistent with the findings of Ito et al. [13], which identified age ≥65 years as a risk factor for elevated blood pressure following mirabegron use. Given that the average age of our study cohort was 65 years, this was likely a factor in the changes in blood pressures observed, although our study sample was too small to demonstrate this relationship.

Mirabegron is contraindicated in patients with uncontrolled hypertension (≥180/110 mmHg), and in the three patients who experienced blood pressure increases, mirabegron was discontinued even though their readings did not reach this threshold. A cross-sectional analysis that utilized data from a multicenter study of atherosclerosis revealed that among patients on antihypertensive medications, OAB was associated with increased systolic blood pressure in men but not in women, while diastolic blood pressure remained consistent across both genders [14]. This emphasizes the importance of studying the use of mirabegron in women with hypertension, as OAB itself should not affect worsening hypertension in women. Future research should explore the benefits and risks of combining mirabegron with antihypertensive medications to optimize management for patients with both OAB and hypertension.

While anticholinergics are the standard treatment for OAB, they can cause severe adverse effects such as constipation, dry mouth, and cognitive dysfunction [15]. The tolerability profile of mirabegron may improve patient adherence to treatment for overactive bladder, as dry mouth is a common reason patients discontinue antimuscarinic therapy. In several large Phase 3 clinical trials, including the well-known CAPRICORN, SCORPIO, ARIES, and Yamaguchi trials, the incidence of dry mouth (1.6-2.8%) with mirabegron was similar to that observed with placebo and significantly less compared with traditional anticholinergics [4]. Mirabegron, hence, offers a valuable alternative, particularly for those who cannot tolerate anticholinergic side effects. However, the relatively high financial cost of mirabegron led to discontinuation in some patients. Only one of these patients successfully transitioned to an OAB alternative, highlighting the need for further research into alternative treatments for those who cannot tolerate either mirabegron or anticholinergics.

Despite its primary indication for OAB, our study suggests that mirabegron was also effective in relieving symptoms of SUI and mixed urinary incontinence (MUI). To our knowledge, evidence for mirabegron's efficacy in pure SUI is limited. Mirabegron has been explored for MUI as part of combination therapies or adjunctive treatments. In some cases, it has been shown to reduce urgency episodes and improve overall bladder compliance when used alongside other interventions like pelvic floor muscle training or midurethral sling surgery. However, standalone efficacy in MUI remains less defined compared to overactive bladder-specific symptoms. Further research is warranted to elucidate the role of mirabegron in the management of SUI. Although SUI is not an approved indication for mirabegron, its potential utility could be considered within the broader framework of individualized treatment planning, particularly in cases of MUI or overlapping symptoms [16].

This study has several limitations, including a small sample size and a focus solely on female patients, which may limit the generalizability of the findings to males. Additionally, as we only examined patients with well-controlled hypertension, the results may not apply to individuals with unstable or labile hemodynamics.

## Conclusions

Our findings show that mirabegron effectively alleviates OAB symptoms, including urgency, urge incontinence, and stress incontinence, and is generally safe and well-tolerated in patients with well-controlled hypertension. However, about 6.5% of patients may still experience elevated blood pressure, highlighting the need for regular blood pressure monitoring, especially in older individuals, with emphasis on home blood pressure monitoring. Mirabegron’s favorable tolerability profile makes it a valuable alternative for patients who struggle with side effects like dry mouth, constipation, and cognitive dysfunction. Despite these advantages, the high cost of the medication poses a significant barrier to long-term adherence. The cost of mirabegron is not subsidized by the national healthcare system as it is not considered a standardized drug. The cost of certain anticholinergics (e.g., oxybutynin and solifenacin) is subsidized as they are considered standardized drugs in our healthcare system. Thus, the cost for mirabegron is 2.39/USD/day, while that of oxybutynin is only 0.5 USD/day. Only one patient successfully transitioned to an alternative OAB therapy, underscoring the need for further research into additional oral treatment options for patients who cannot tolerate either mirabegron or anticholinergics. While mirabegron offers an effective and generally safe alternative for managing OAB, particularly for patients with hypertension, careful blood pressure monitoring is key to mitigate potential risks. Further studies with larger, more diverse populations are needed to confirm these findings and explore cost-effective treatment strategies for patients facing challenges with existing therapies.

## References

[1] Akbar A, Liu K, Michos ED (2022). Association of overactive bladder with hypertension and blood pressure control: the Multi-Ethnic Study of Atherosclerosis (MESA). Am J Hypertens.

[2] Chapple CR, Cardozo L, Nitti VW, Siddiqui E, Michel MC (2014). Mirabegron in overactive bladder: a review of efficacy, safety, and tolerability. Neurourol Urodyn.

[3] Chuang YC, Liu SP, Lee KS (2019). Prevalence of overactive bladder in China, Taiwan, and South Korea: results from a cross-sectional, population-based study. Low Urin Tract Symptoms.

[4] Fuchs SC, Mello RG, Fuchs FC (2013). Home blood pressure monitoring is a better predictor of cardiovascular disease and target organ damage than office blood pressure: a systematic review and meta-analysis. Curr Cardiol Rep.

[5] Heintjes EM, Bezemer ID, Prieto-Alhambra D (2020). Evaluating the effectiveness of an additional risk minimization measure to reduce the risk of prescribing mirabegron to patients with severe uncontrolled hypertension in four European countries. Clin Epidemiol.

[6] Hiriscau EI, Buzdugan EC, Hui LA, Bodolea C (2022). Exploring the relationship between frailty, functional status, polypharmacy, and quality of life in elderly and middle-aged patients with cardiovascular diseases: a one-year follow-up study. Int J Environ Res Public Health.

[7] Irwin DE, Kopp ZS, Agatep B, Milsom I, Abrams P (2011). Worldwide prevalence estimates of lower urinary tract symptoms, overactive bladder, urinary incontinence and bladder outlet obstruction. BJU Int.

[8] Irwin DE, Milsom I, Hunskaar S (2006). Population-based survey of urinary incontinence, overactive bladder, and other lower urinary tract symptoms in five countries: results of the EPIC study. Eur Urol.

[9] Ito H, Matsuo T, Mitsunari K, Ohba K, Miyata Y (2022). Impact of mirabegron administration on the blood pressure and pulse rate in patients with overactive bladder. Medicina (Kaunas).

[10] Lozano-Ortega G, Walker DR, Johnston K (2020). Comparative safety and efficacy of treatments for overactive bladder among older adults: a network meta-analysis. Drugs Aging.

[11] Makhani A, Thake M, Gibson W (2020). Mirabegron in the treatment of overactive bladder: safety and efficacy in the very elderly patient. Clin Interv Aging.

[12] Matta R, Saskin R, Neu S, Locke JA, Kowalczyk A, Steup A, Herschorn S (2023). Predicting mirabegron treatment response in patients with overactive bladder: a post hoc analysis of data from clinical trials. Eur Urol Focus.

[13] Myint PK, Fox C, Kwok CS, Luben RN, Wareham NJ, Khaw KT (2015). Total anticholinergic burden and risk of mortality and cardiovascular disease over 10 years in 21,636 middle-aged and older men and women of EPIC-Norfolk prospective population study. Age Ageing.

[14] O'Kane M, Robinson D, Cardozo L, Wagg A, Abrams P (2022). Mirabegron in the management of overactive bladder syndrome. Int J Womens Health.

[15] Takahashi S, Mishima Y, Kuroishi K, Ukai M (2022). Efficacy of mirabegron, a β(3)-adrenoreceptor agonist, in Japanese women with overactive bladder and either urgency urinary incontinence or mixed urinary incontinence: post-hoc analysis of pooled data from two randomized, placebo-controlled, double-blind studies. Int J Urol.

[16] Warren K, Burden H, Abrams P (2016). Mirabegron in overactive bladder patients: efficacy review and update on drug safety. Ther Adv Drug Saf.

